# A Novel Nomogram to Predict Prolonged Survival After Hepatectomy in Repeat Recurrent Hepatocellular Carcinoma

**DOI:** 10.3389/fonc.2021.646638

**Published:** 2021-03-25

**Authors:** Qiongxuan Fang, Ruifeng Yang, Dongbo Chen, Ran Fei, Pu Chen, Kangjian Deng, Jie Gao, Weijia Liao, Hongsong Chen

**Affiliations:** ^1^Beijing Key Laboratory of Hepatitis C and Immunotherapy for Liver Diseases, Peking University Hepatology Institute, Peking University People's Hospital, Beijing, China; ^2^Laboratory of Hepatobiliary and Pancreatic Surgery, Affiliated Hospital of Guilin Medical University, Guilin, China; ^3^Department of Hepatobiliary Surgery, Peking University People's Hospital, Beijing, China

**Keywords:** nomogram, post-recurrence survival, prognosis, TNM stage, BCLC stage

## Abstract

**Background:** Repeat hepatectomy is an important treatment for patients with repeat recurrent hepatocellular carcinoma (HCC).

**Methods:** This study was a multicenter retrospective analysis of 1,135 patients who underwent primary curative liver resection for HCC. One hundred recurrent patients with second hepatectomy were included to develop a nomogram to predict the risk of post-recurrence survival (PRS). Thirty-eight patients in another institution were used to externally validate the nomogram. Univariate and multivariate Cox regression analyses were used to identify independent risk factors of PRS. Discrimination, calibration, and the Kaplan–Meier curves were used to evaluate the model performance.

**Results:** The nomogram was based on variables associated with PRS after HCC recurrence, including the tumor, node, and metastasis (TNM) stage; albumin and aspartate aminotransferase levels at recurrence; tumor size, site, differentiation of recurrences; and time to recurrence (TTR). The discriminative ability of the nomogram, as indicated by the C statistics (0.758 and 0.811 for training cohort and external validation cohorts, respectively), was shown, which was better than that of the TNM staging system (0.609 and 0.609, respectively). The calibration curves showed ideal agreement between the prediction and the real observations. The area under the curves (AUCs) of the training cohort and external validation cohorts were 0.843 and 0.890, respectively. The Kaplan–Meier curve of the established nomogram also performed better than those of both the TNM and the BCLC staging systems.

**Conclusions:** We constructed a nomogram to predict PRS in patients with repeat hepatectomy (RH) after repeat recurrence of HCC.

## Introduction

Hepatocellular carcinoma (HCC) recurrence is a common phenomenon after resection in patients with preserved liver function reserve. The 5-year HCC recurrence rate after curative resection is over 50% (1, 2); and, of all recurrence patterns, the most frequent is intrahepatic recurrence (3, 4). However, there is little agreement on the criteria for a standardized treatment strategy for recurrent HCC. Repeated hepatectomy (RH) is one of the important treatments for repeat recurrence HCC. Aggressive treatment of HCC recurrence after liver resection is related to prolonged overall survival (OS) (5, 6). Faber et al. (7) retrospectively studied 27 patients to clarify the safety and effectiveness of RH as a curative option for intrahepatic HCC recurrence. Chan et al. (8) evaluated the efficacy of salvage liver transplantation (SLT), RH, and repeated radiofrequency ablation (rRFA) in patients with post-operative HCC recurrence and found that SLT and RH led to comparable survival outcomes and that both treatments were better than rRFA.

However, RH is not indicated for patients with impaired liver function and multifocal intrahepatic or extrahepatic recurrence. A previous study reported the use of a nomogram to predict prognosis after the second hepatectomy (9). Given that the nomogram did not include factors related to impaired liver function, clinical physicians had to further consider these factors, but they could not determine the relative importance of these factors in prognosis. Moreover, there were no external validation cohorts to validate the nomogram.

Post-recurrence survival (PRS) of patients with HCC is greatly impacted by features of recurrence rather than by features of the primary tumors, and it could also be convenient for clinical physicians to evaluate the survival time for patients who received the second operation. The purpose of this study was to identify the clinical and pathological characteristics associated with PRS after RH in patients with HCC. For patients from the Affiliated Hospital of Guilin Medical College (Guilin cohort), we first used the information of 100 patients who underwent a second hepatectomy for recurrent HCC to construct a nomogram to predict the individual risk of PRS after initial recurrence. We validated the nomogram with 38 patients who underwent a second hepatectomy for recurrent HCC from the external Peking University People's Hospital (PKUPH) cohort.

## Materials and Methods

### Patient Population and Data Collection

Between September 27, 1995 and December 31, 2016, data on consecutive patients with primary HCC who underwent curative liver resection were prospectively collected at the Affiliated Hospital of Guilin Medical University, China. Between December 2005 and December 2019, data on consecutive patients with primary HCC who underwent curative liver resection were prospectively collected at PKUPH, China. The Institutional Ethics Committee of the Affiliated Hospital of Guilin Medical College and PKUPH approved this study, which follows the ethical guidelines of the 2013 Declaration of Helsinki. Due to the long-term of the study, we proceeded to inform the patients before the surgical treatment and provided them with the informed consent form to sign.

The inclusion criteria were as follows: (1) Patients who underwent R0 liver resection; (2) patients with no evidence of extrahepatic metastasis or macroscopic tumor thrombus in the major portal/hepatic vein and biliary tract before the primary and repeat hepatectomies; (3) patients who were not receiving adjuvant treatment; and (4) patients with Child-Pugh A or B liver function. The exclusion criteria were as follows: (1) Patients who had received any preoperative or post-operative anticancer treatments; (2) patients who had a history of other cancers or had incomplete clinical data; and (3) patients who died within 30 days of operation (to avoid the inclusion of deaths due to post-operative complications). We used the data from patients at PKUPH (*n* = 362) between 2005 and 2019 as an external validation cohort using the same inclusion and exclusion criteria.

Demographic and clinicopathological data of the patients with primary recurrences were collected, including age, sex, family history, alcohol history, liver function, blood routine examination, and serum a-fetoprotein (AFP) tests, lymph node metastasis, tumor size, tumor number, tumor site (caudate lobe, left, or right), tumor differentiation (i.e., low, median or high), time to recurrence (TTR), and the TNM stage of the disease. Tumor size was defined as the sum of the diameters of all the resected tumors. The final pathological outcomes were used to evaluate the resection margin status (negative [R0]) and the lymph node status (no metastasis[N0] or lymph node metastasis [N1]). The primary outcomes of interest were PRS.

### Follow-Up

After curative liver resection, patients received regular medical follow-ups every 2 months for the first 2 years and every 3–6 months thereafter. At each follow-up visit, patients had a routine examination of the physical checkup, determination of serum AFP levels, liver function tests, and at least one imaging examination, including abdominal ultrasound, contrast-enhanced CT scan, or MRI. For patients who were suspected of having HCC recurrence based on liver ultrasound or dynamically elevated AFP levels, either a contrast-enhanced CT scan or MRI was carried out to confirm or exclude the diagnosis. Chest CT was annually performed to exclude lung metastasis. Each recurrence time was defined as the date of the first positive imaging examination result. PRS was defined as the interval from the time of the primary recurrence to the final follow-up date or the time of patient death.

### Statistical Analysis

Categorical variables were presented as whole numbers and proportions and were compared using the χ^2^ test. Continuous variables were presented as medians with interquartile ranges (IQRs) and compared using an unpaired, two-tailed *t*-test or the Mann–Whitney test. The Mann–Whitney test was used when normal distribution and homogeneity of variance could not meet the requirements of the *t*-test. Survival curves were compared using the Kaplan–Meier method and the log-rank test. Clinicopathological variables were considered discrete and were converted to categorical variables based on the clinical importance and were identified predictors according to previously published studies (9, 10).

We performed univariate and multivariate Cox regression analyses to confirm independent prognostic factors of PRS. Variables with statistically significant *P*-values on univariate analysis were selected into the multivariate Cox proportional hazard regression model. Backward stepwise selection with the Akaike information criterion (AIC) was applied to select the independent significant variables used in the development of the nomogram. The variation inflation factor was used to evaluate multicollinearity, and no significant interaction was found. Hazard ratios (HRs) of the variables were shown with their 95% CIS (11). The model performance was evaluated internally and externally by discrimination and calibration *via* the Harrell's concordance index (C-index) (12). Finally, the Kaplan–Meier curves were plotted with the tertiles of patients layered on the scores predicted by the established nomogram to further evaluate calibration. The model was validated by bootstrapping with a resampling of 1,000 to quantify any overfitting of the modeling strategy. All the statistical analyses were performed using the R software version 3.6.1 (www.r-project.org). *P*-values lower than 0.05 were considered statistically significant, and all tests were two-sided.

## Results

### Clinicopathological Characteristics

**Figure 1:**
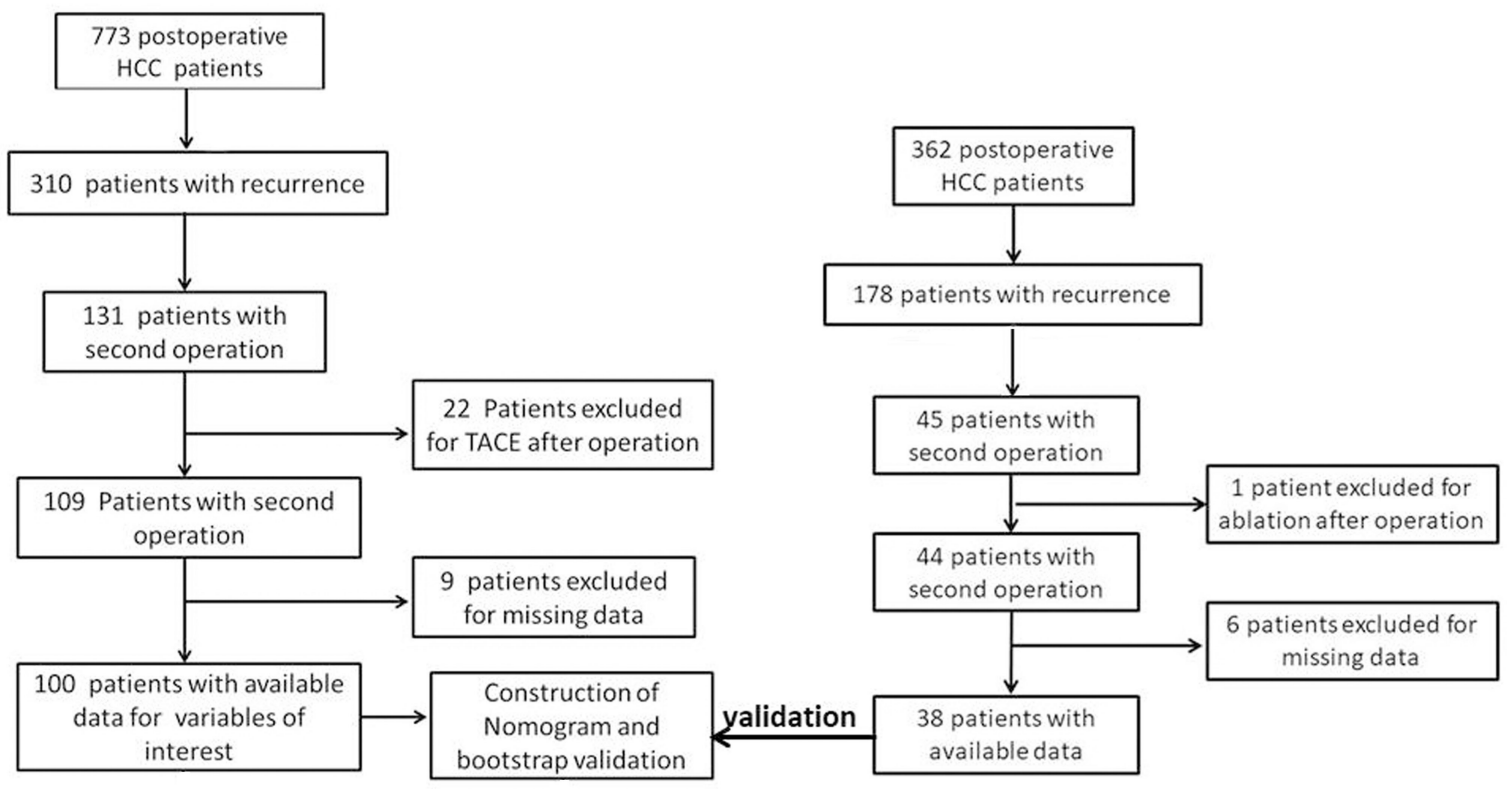
Flowchart of this study.

The flowchart of patient recruitment is shown in Figure 1. In the Guilin cohort, 773 post-operative patients were enrolled for follow-up; of these, 294 patients recurred after the primary surgery, 131 recurrent patients received the second hepatectomy, 22 patients were excluded because they received transarterial chemoembolization (TACE) treatment after hepatectomy, nine patients were lost to follow-up, and finally 100 patients were identified as training cohort; In the PKUPH cohort, 362 post-operative patients were enrolled for follow-up; of these, 178 patients recurred after the primary surgery, 45 recurrent patients received the second hepatectomy, 1 patient was excluded because they received the ablation treatment after hepatectomy, 6 patients were lost to follow-up, and finally 38 patients were identified as the external validation cohort. The clinical and pathological characteristics of patients with HCC in the training cohort (*n* = 100) and external validation cohort are summarized in (Supplementary Table 1). There were no differences in baseline indicators between the training cohort and the external validation cohorts, except in factors such as age, WBC count, LYMPH count, NEUT count, ALT and AST level, tumor difference, and HBsAg level. For the Guilin cohort, the median follow-up time was 34.2 months (range 19.7–56.5). About 38.1% (294 of 773) of the patients had a first recurrence of the disease, and 26% (26 of 100) of the patients had re-recurrence. The 2- and 5-year PRS rates were 57 and 15%, respectively, and the median PRS was 27.5 months.

### Independent Prognostic Factors in the Training Cohort

All variables listed in Supplementary Table 1 were used for univariate analysis, which was shown in Table 1 and Supplementary Table 2. Variables with *P* < 0.05 were used in the multivariate Cox regression analysis. Multivariate analysis revealed that the TNM stage, tumor site, tumor size, tumor differentiation, albumin (ALB) and aspartate aminotransferase (AST) levels, and TTR time were the seven independent prognostic factors for PRS (*P* < 0.05) (Table 1).

**Table 1:** Cox proportional hazards regression model showing the association of variables with post-recurrence survival.

	**Guilin cohort (*n =* 100)**			
	**Univariable**		**Multivariable**	
**Variable**	**HR (95% CI)**	***P-*value**	**HR (95% CI)**	***P-*value**
**Factors selected**				
TNM stage				
I	1 [Reference]		1 [Reference]	
II	2.22 (1.13–4.36)	0.02	2.82 (1.17–6.77)	0.02
III	3.93 (1.86–8.32)	<0.001	2.76 (1.08–7.04)	0.03
Differentiation				
Low	1 [Reference]		1 [Reference]	
High	0.24 (0.08–0.67)	<0.01	0.19 (0.06–0.61)	<0.01
Median	0.59 (0.23–1.09)	0.09	0.35 (0.17–0.73)	<0.01
Tumor site				
Caudate lobe	1 [Reference]		1 [Reference]	
Left	0.35 (0.08–1.53)	0.16	0.14 (0.03–0.74)	0.02
Right	0.21 (0.05–0.91)	0.03	0.05 (0.01–0.31)	<0.001
Diameter, cm				
< =5	1 [Reference]		1 [Reference]	
>5	2.68 (1.45–4.97)	<0.01	1.39 (0.66–2.91)	0.05
ALB (g/L)				
<28	1 [Reference]		1 [Reference]	
(28, 35)	0.008 (0.0004–0.15)	<0.01	0.003 (0.0002–0.07)	<0.001
>=35	0.007 (0.0004–0.11)	<0.001	0.002 (0.0001–0.04)	<0.001
AST (IU/L)				
< =40	1 [Reference]		1 [Reference]	
>40	2.36 (1.36–4.11)	<0.01	2.26 (1.07–4.77)	0.03
TTR (month)				
<=12	1 [Reference]		1 [Reference]	
>12	0.447 (0.26–0.76)	<0.01	0.38 (0.21–0.68)	<0.01

### Prognostic Nomogram for PRS

**Figure 2:**
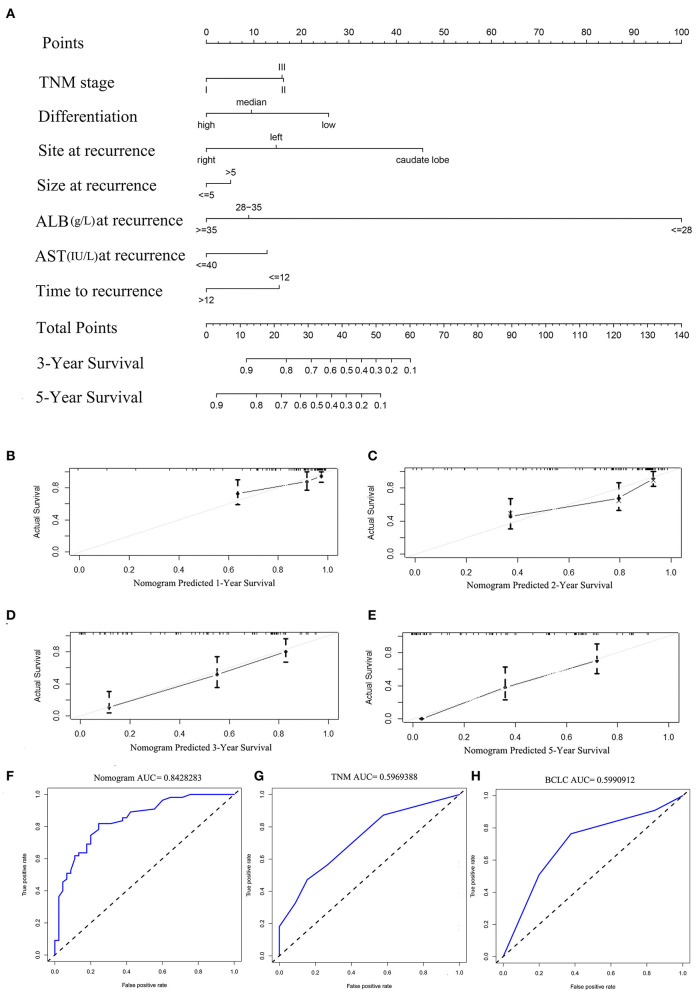
**(A)** Nomogram for predicting the 3- and 5-year post-recurrence survival rates in patients with repeat hepatectomy (RH) of hepatocellular carcinoma (HCC). The calibration curve for predicting the 1-, 2-, 3-, and 5- year post-recurrence survival in the training **(B–E)** cohort; nomogram-predicted probability of PRS is plotted on the x-axis; actual PRS is plotted on the y-axis. The area under the curve (AUC) of the nomogram, and the tumor, node, and metastasis (TNM) and BCLC stage systems in the training cohort **(F–H)**.

The prognostic nomogram for predicting PRS of the recurrent patients after RH is shown in Figure 2A. The nomogram was constructed based upon the following seven independent prognostic factors identified in the Cox model: TNM stage (I, II, or III), tumor site (caudate lobe, left, or right), tumor size (≤5 or >5 cm), tumor differentiation (low, high, or median), ALB (<28, 28–35 or ≥35 g/L), AST (≤40 or >40 IU/L), and TTR (≤12 or >12 months). The nomogram was then used to predict 3- and 5-year PRS rates for recurrent patients after RH of HCC (Figure 2A). Each individual can be assigned a mortality risk by adding seven individual scores identified in the nomogram; the higher total scores are associated with a worse prognosis.

### Discriminative Ability of the Prognostic Nomogram

**Figure 3:**
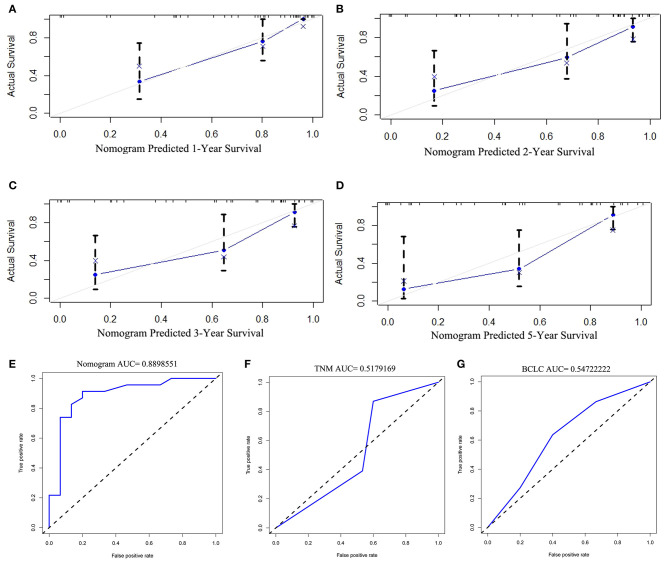
The calibration curve for predicting the 1-, 2-, 3-, and 5- year post-recurrence survival in the training **(A–D)** cohort; nomogram-predicted probability of PRS is plotted on the x-axis; actual PRS is plotted on the y-axis. The AUC of the nomogram, and the TNM and BCLC stage systems in external validation cohorts **(E–G)**.

The discriminative ability of the PRS prediction model by C statistics was 0.758 (95% CI, 0.685–0.831), which is better than the TNM staging system of recurrence (0.609, 95% CI, 0.535–0.683, *P* < 0.01). The prediction of the 1-, 2-, 3-, and 5-year PRS rates by the 33-sample bootstrapped calibration plot are shown in Figures 2B–E, demonstrating an ideal agreement between nomogram prediction and real observations. The generated model was internally validated by the bootstrap validation method with 1,000 resamplings (the C statistics was 0.703). For the external validation cohort, the C statistics was 0.811 (95% CI, 0.762–0.860), which is better than the TNM staging system of recurrence (0.609, 95% CI, 0.546–0.672, *P* < 0.01). The prediction of the 1-, 2-, 3-, and 5-year PRS rates by the 12-sample bootstrapped calibration plot were shown in Figures 3A–D, demonstrating an ideal agreement between nomogram prediction and real observations. The AUCs of the training cohort and external validation cohorts were 0.843 and 0.890, respectively, which were better than those of the TNM and BCLC stage systems as shown in Figures 2F–H and Figures 3E–G.

**Figure 4:**
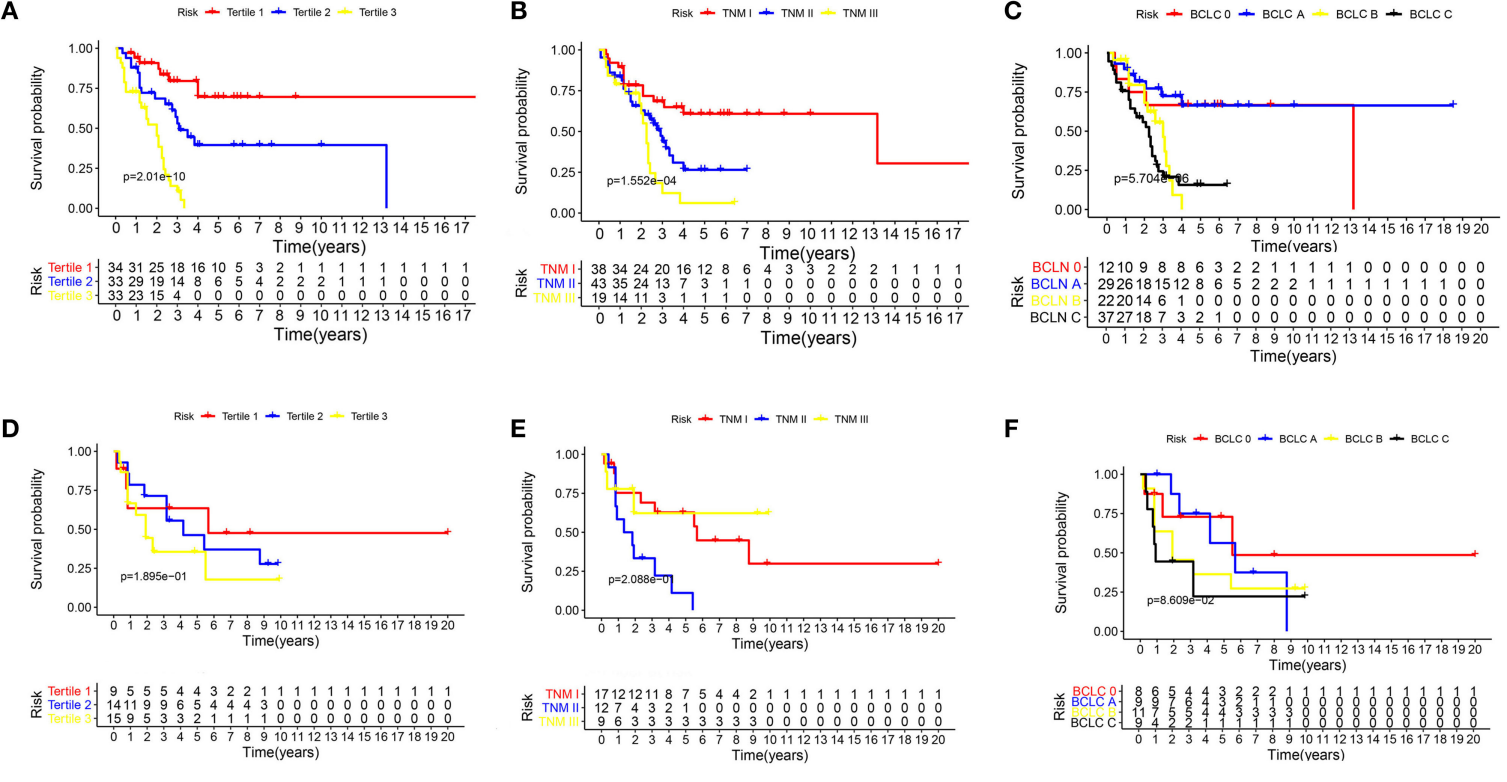
The Kaplan–Meier curves for subgroups of patients. Patients were stratified by the prognostic score **(A)**, TNM stage **(B)**, and BCLC stage **(C)** in the training cohort. Patients were stratified by the prognostic score **(D)**, TNM stage **(E)**, and BCLC stage **(F)** in the external validation cohorts.

The Kaplan–Meier curves were plotted to further verify the power of the nomogram in predicting PRS (Figures 4A,D). In the training cohort, the nomogram stratified patients into low- (total score ≤ 27), medium- (total score 27–47), and high-risk (total score > 47) subgroups. Patients in the high-risk group (tertile 3) had a worse outcome (0% 5-year PRS) in comparison with patients in the low-risk group (tertile 1) and the median-risk group (tertile 2) (69.6 and 39.5% 5-year PRS, respectively) (*P* < 0.001), meanwhile, the predicted 2-year PRS rates in low-, median-, and high-risk groups were 87.2, 68.6, and 45.4%, respectively (*P* < 0.001) (Figure 4A). The Kaplan–Meier curves were also constructed for the TNM and BCLC staging systems of both the training and validation groups (Figures 4B,C,E,F). There was an overlap of curves for patients in TNM stage I, II, and III of the training cohort during the first 2 years of survival (Figure 4B), and patients in the BCLC stage had a similar result (Figure 4C). In the external validation cohort, the Kaplan–Meier curve of the established nomogram (Figure 4D) also performed better than those of both the TNM (Figure 4E) and BCLC (Figure 4F) staging system.

## Discussion

In the current study, we constructed a novel nomogram to predict PRS in recurrent patients after RH in HCC and then externally validated patients in the PKUPH cohort. This clinical context occurs in patients treated with RH without other treatments.

Repeat hepatectomy is reported to prolong the OS time for patients with recurrent HCC after liver resection (5, 13–15). A previous study established a nomogram to predict the survival of patients with recurrence of HCC after the primary operation and identified repeat resection as an independent prognostic factor associated with prolonged survival, but it also included patients managed with other treatments, including molecular targeted therapy, systematic chemotherapy, radiotherapy, and supportive care (10), so this nomogram is not particularly helpful. For example, RH treatment is scored zero in the nomogram, which means patients who select RH have a better prognosis than those who select other treatments. However, RH is not fit for all recurrent patients. In our opinion, all treatments should have their own standards for evaluation. Clinical physicians could compare the outcomes based on these standards and select the optimal treatment for patients with recurrent HCC.

Another particular strength of this study is that the nomogram included a wide array of variables (TNM stage, liver function, tumor characteristics, and TTR) identified in previous publications as being related to prognosis after liver resection of the HCC (10, 13, 14, 16). Zou et al. (9) previously developed a nomogram among patients with RH of HCC that incorporated TTR, HBV-DNA level, and tumor characteristics at the initial surgery in the model, having identified TTR as the most effective predictive factor for mortality. The inclusion of risk factors without liver function is problematic because it is self-explanatory that liver function is closely related to prognosis (17). Our study incorporated all the variables collected at recurrences, and both serum AST and ALB are included in the liver function tests, which implies that the liver function has been taken into account in our model. It is worth mentioning that serum ALB levels occupy the most important position in the nomogram. Some studies (18–20) have reported that tumor differentiation and tumor size are associated with disease-free survival and/or OS after the initial surgery. In contrast, other researchers have put forward that there is no correlation between these tumor characteristics and disease-free survival and/or OS (21). In the present study, we found a significant association not only between tumor differentiation and tumor size but also proved that tumor site (left, right, or caudate lobe) is correlated with PRS, the hazard ratio being shown in Table 1. Because of a specific anatomical characteristic, tumors on the caudate lobe are difficult to completely resect (22), which was first identified as the independent risk factor in predicting PRS in this study. Furthermore, we also noted that TTR is strongly linked to outcomes. A previous study has identified 2 years after resection as the optimal cutoff value to distinguish late recurrence from early recurrence (23), while this study implies that <2 year from repeat resection to recurrence has a close correlation with PRS, which is in accordance with nomograms in other studies (9, 10).

Accurate risk stratification of the patients with post-operative recurrence in HCC is essential because the prognosis of patients may vary (24). However, the TNM staging system was less useful than the nomogram developed in this study for PRS prediction in both the training cohorty and external validation cohorts (C-index, 0.758 vs. 0.609, 0.811 vs. 0.609, respectively), which suggests that our nomogram has the ability to predict post-operative survival after recurrence. Indeed, when stratified into tertiles in the survival analysis, the established nomogram could identify subgroups of patients who were at different risks of death in both the training cohort and validation cohorts.

In conclusion, we constructed a nomogram to predict PRS in patients with RH after the recurrence of HCC. The nomogram performed well on external validation cohort. This study also has limitations. The number of patients who accepted a second radical surgery is small, although the clinical and pathological data were collected in two centers. The sample size is still small, and more studies are needed to externally validate the established nomogram. In addition, imaging data were not collected; therefore, the nomogram could not evaluate its effect.

## Data Availability Statement

The original contributions generated for the study are included in the article/Supplementary Material, further inquiries can be directed to the corresponding author/s.

## Ethics Statement

The studies involving human participants were reviewed and approved by Peking University People's Hospital Affiliated Hospital of Guilin Medical University. The patients/participants provided their written informed consent to participate in this study.

## Author Contributions

HC, QF, WL, and JG designed the study. QF analyzed the data and wrote the manuscript. RY, DC, and RF provided technical expertise and support. PC and KD collected the clinical information and scheduled the follow-up plan. All authors contributed to the article and approved the submitted version.

## Conflict of Interest

The authors declare that the research was conducted in the absence of any commercial or financial relationships that could be construed as a potential conflict of interest.

## Abbreviations

PRS: post-recurrence survival
RH: repeat hepatectomy
TTR: time to recurrence
HCC: hepatocellular carcinoma.
