# Supplementary material for: A Novel Nomogram to Predict Prolonged Survival After Hepatectomy in Repeat Recurrent Hepatocellular Carcinoma
Source: Front Oncol. 2021 Mar 25;11:646638. doi: 10.3389/fonc.2021.646638 (PMC8027067; doi:10.3389/fonc.2021.646638)
Supplement: Supplementary file 1 [file Table_1.DOCX]

| Table S1. Baseline information and tumor characteristics of the patients undergoing repeat surgical resection for HCC. | | | |
| --- | --- | --- | --- |
| Characteristic | Training cohort | External validation cohort | *P* |
|  | (Guilin cohort, n=100) | (PKUPH cohort, n=38) | Value^a^ |
| Age, yr, median | 51.0(46.0-58.0) | 62.0(52.0-64.0) | <0.01 |
| (IQR) |  |  |  |
| WBC *109/L | 5.44(4.14-6.85) | 6.37(4.91-8.30) | <0.01 |
| PLT *109/L, | 144(99.5-189.25) | 104(84.1-150.8) | 0.06 |
| median |  |  |  |
| LYMPH# *109/L | 1.61(1.25-1.99) | 1.89(1.03-10.78) | <0.01 |
| NEUT# *109/L | 2.99(2.3-4.24) | 7.93(3.55-72.85) | <0.01 |
| ALBI | -2.76(-3.05~-2.46) | -2.49(-2.90~-1.98) | 0.1 |
| TB, umol/L | 12.39(8.86-15.68) | 16.35(12.08-19.36) | 0.96 |
| DB, umol/L | 4.66(3.02-5.56) | 6.5(4.18-8.9) | 0.63 |
| ALB, g/L | 40.36(37.69-44.27) | 37.65(33.38-42.63) | 0.28 |
| A/G | 1.37(1.19-1.56) | 1.59(1.35-1.83) | 0.002 |
| ALP, IU/L | 77.2(66.42-92.89) | 72(56.25-113.75) | 0.25 |
| GGT, IU/L | 48.05(28.62-105.57) | 47.5(35.75-62.25) | 0.11 |
| ALT, IU/L | 27.98(17.2-44.89) | 32(22-93) | 0.02 |
| AST, IU/L | 32.8(23.55-44.98) | 30(22-74) | <0.01 |
| AFP, ng/mL | 28.26(4.27-617.3) | 11.77(3.87-103) | 0.18 |
| Sex |  |  | 0.38 |
| Female | 16 (16%) | 7(19.4%) |  |
| Male | 84(84%) | 31(81.6%) |  |
| Family history | |  | 0.23 |
| NO | 81(81%) | 33(86.8%) |  |
| YES | 19(19%) | 5(13.2%) |  |
| Alcohol history | |  | 0.11 |
| NO | 76(76%) | 29(76.3%) |  |
| YES | 24(24%) | 9(23.7%) |  |
| Tumor location | |  |  |
| caudate lobe | 2(2%) | 0 | 0.22 |
| left | 30(30%) | 19(50%) |  |
| right | 68(68%) | 14(36.8%) |  |
| Tumor number | |  |  |
| single | 70(70%) | 24(63.2%) | 0.43 |
| multiple | 30(30%) | 14(36.8%) |  |
| Tumor diameter |  |  |  |
| (cm) |  |  |  |
| <=5 | 66(66%) | 21(55.3%) | 0.23 |
| >5 | 34(34%) | 17(44.7%) |  |
| TNM stage | |  |  |
| I | 38(38%) | 17(44.7%) | 0.94 |
| II | 43(43%) | 12(31.6%) |  |
| III | 19(19%) | 11(28.9%) |  |
| Difference |  |  |  |
| low | 28(28%) | 4(10.5%) | 0.003 |
| high | 16(16%) | 3(7.9%) |  |
| median | 56(56%) | 31(81.6) |  |
| Child-Paugh grade | |  |  |
| A | 96(96%) | 35(92.1%) | 0.33 |
| B | 4(4%) | 3(7.9%) |  |
| PVTT |  |  |  |
| NO | 76(76%) | 30(78.9%) | 0.7 |
| YES | 24(24%) | 8(21.1) |  |
| HBsAg |  |  |  |
| Negative | 19(19%) | 18(47.4%) | 0.001 |
| Positive | 81(81%) | 20(52.6%) |  |
| HBeAg |  |  | 0.528 |
| Negative | 97(97) | 36(94.7%) |  |
| Positive | 3(3%) | 2(5.3%) |  |
| HBeAb |  |  | 0.638 |
| Negative | 20(20%) | 9(23.7) |  |
| Positive | 80(80%) | 29(76.3%) |  |
| HBcAb |  |  |  |
| Negative | 5(5%) | 2(5.3%) | 0.95 |
| Positive | 95(95%) | 36(94.7%) |  |
| Time to recurrence |  |  | 0.02 |
| (month) |  |  |  |
| <=12 | 38(38%) | 7(18.4%) |  |
| >12 | 62(62%) | 31(81.6%) |  |
| Survival state | |  | 0.55 |
| Alive | 45(45%) | 15(39.5%) |  |
| Dead | 55(55%) | 23(60.5%) |  |
| *P* Value^a^: t Test comparision between training cohort and external validation cohort. | | | |
