# Supplementary material for: A Novel Nomogram to Predict Prolonged Survival After Hepatectomy in Repeat Recurrent Hepatocellular Carcinoma
Source: Front Oncol. 2021 Mar 25;11:646638. doi: 10.3389/fonc.2021.646638 (PMC8027067; doi:10.3389/fonc.2021.646638)
Supplement: Supplementary file 2 [file Table_2.DOCX]

|  | **Univariable** | | **Multivariable** | |
| --- | --- | --- | --- | --- |
| **Variable** | **HR (95% CI)** | **P Value** | **HR (95% CI)** | **P Value** |
| **Factors Not Selected** | |  |  |  |
| Family |  |  |  |  |
| NO | 1 [Reference] | |  |  |
| YES | 0.24(0.09-0.61) | 0.002 | 0.6(0.26-1.41) | 0.24 |
| GGT(IU/L) |  |  |  |  |
| <=100 | 1 [Reference] | |  |  |
| >100 | 1.85(1.05-3.25) | 0.03 | 1.01(0.55-1.86) | 0.97 |
| AFP (ng/mL) |  |  |  |  |
| <20 | 1 [Reference] | |  |  |
| [20,400) | 1.67(0.84-3.31) | 0.14 | 1.39(0.76-2.53) | 0.29 |
| >=400 | 2.07(1.11-3.87) | 0.02 | 1.01(0.55-1.74) | 0.94 |
| ALBI |  |  |  |  |
| <=-2.6 | 1 [Reference] | |  |  |
| >-2.6 | 1.34(0.74-2.39) | 0.33 | NA | NA |
| Age |  |  |  |  |
| <=50 | 1 [Reference] | |  |  |
| >50 | 0.69(0.41-1.18) | 0.17 | NA | NA |
| Gender |  |  |  |  |
| FEMALE | 1 [Reference] | |  |  |
| MALE | 1.05(0.51-2.14) | 0.9 | NA | NA |
| Alcohol |  |  |  |  |
| NO | 1 [Reference] | |  |  |
| YES | 0.74(0.38-1.45) | 0.38 | NA | NA |
| Tumor number |  |  |  |  |
| Single | 1 [Reference] | |  |  |
| Multiple | 1.66(0.95-2.89) | 0.07 | NA | NA |
| PVTT |  |  |  |  |
| NO | 1 [Reference] | |  |  |
| YES | 1.51(0.83-2.75) | 0.18 | NA | NA |
| WBC (*109/L) |  |  |  |  |
| <=4 | 1 [Reference] | |  |  |
| (4,10] | 0.99(0.51-1.94) | 0.99 | NA | NA |
| >10 | 4.68(0.57-37.97) | 0.14 | NA | NA |
| PLT (*109/L) |  |  |  |  |
| <100 | 1 [Reference] | |  |  |
| [100-300] | 1.11(0.57-2.16) | 0.77 | NA | NA |
| >300 | 0.79(0.23-2.83) | 0.72 | NA | NA |
| LYMPH (*109/L) |  |  |  |  |
| <1.1 | 1 [Reference] | |  |  |
| >=1.1 | 1.16(0.58-2.3) | 0.68 | NA | NA |
| NEUT (*109/L) |  |  |  |  |
| <1.8 | 1 [Reference] | |  |  |
| [1.8-6.3) | 1.56(0.62-3.93) | 0.35 | NA | NA |
| >=6.3 | 2.17(0.52-9.09) | 0.29 | NA | NA |
| TB (umol/L) |  |  |  |  |
| <=17.1 | 1 [Reference] | |  |  |
| >17.1 | 1.22(0.63-2.36) | 0.56 | NA | NA |
| DB (umol/L) |  |  |  |  |
| <=6.8 | 1 [Reference] | |  |  |
| >6.8 | 1.48(0.69-3.14) | 0.31 | NA | NA |
| A/G |  |  |  |  |
| <=1.5 | 1 [Reference] | |  |  |
| >1.5 | 1.04(0.59-1.82) | 0.89 | NA | NA |
| ALT(IU/L) |  |  |  |  |
| <=44 | 1 [Reference] | |  |  |
| >44 | 1.86(0.93-3.69) | 0.08 | NA | NA |
| Recurrence pattern |  |  |  |  |
| Ipsilateral | 1 [Reference] | |  |  |
| Contralateral | 0.56(0.30-1.02) | 0.06 | NA | NA |
| HBsAg |  |  |  |  |
| Negative | 1 [Reference] | |  |  |
| Positive | 0.98(0.51-1.91) | 0.96 | NA | NA |
